# Comparative genomic analysis revealed genetic divergence between *Bifidobacterium catenulatum* subspecies present in infant versus adult guts

**DOI:** 10.1186/s12866-022-02573-3

**Published:** 2022-06-16

**Authors:** Jiaqi Liu, Weicheng Li, Caiqing Yao, Jie Yu, Heping Zhang

**Affiliations:** Key Laboratory of Dairy Biotechnology and Engineering (Inner Mongolia Agricultural University), Ministry of Education; Key Laboratory of Dairy Products Processing, Ministry of Agriculture and Rural Affairs; Inner Mongolia Key Laboratory of Dairy Biotechnology and Engineering, Inner Mongolia Agricultural University, Hohhot, China

**Keywords:** *Bifidobacterium catenulatum*, Genomics, Carbohydrate utilization, Plant-derived glycan, Human milk oligosaccharides

## Abstract

**Background:**

The two subspecies of *Bifidobacterium catenulatum*, *B. catenulatum* subsp. *kashiwanohense* and *B. catenulatum* subsp. *catenulatum*, are usually from the infant and adult gut, respectively. However, the genomic analysis of their functional difference and genetic divergence has been rare. Here, 16 *B. catenulatum* strains, including 2 newly sequenced strains, were analysed through comparative genomics.

**Results:**

A phylogenetic tree based on 785 core genes indicated that the two subspecies of *B. catenulatum* were significantly separated. The comparison of genomic characteristics revealed that the two subspecies had significantly different genomic sizes (*p* < 0.05) but similar GC contents. The functional comparison revealed the most significant difference in genes of carbohydrate utilisation. Carbohydrate-active enzymes (CAZyme) present two clustering patterns in *B. catenulatum.* The *B. catenulatum* subsp. *kashiwanohense* specially including the glycoside hydrolases 95 (GH95) and carbohydrate-binding modules 51 (CBM51) families involved in the metabolism of human milk oligosaccharides (HMO) common in infants, also, the corresponding fucosylated HMO gene clusters were detected. Meanwhile, *B. catenulatum* subsp. *catenulatum* rich in GH3 may metabolise more plant-derived glycan in the adult intestine.

**Conclusions:**

These findings provide genomic evidence of carbohydrate utilisation bias, which may be a key cause of the genetic divergence of two *B. catenulatum* subspecies.

**Supplementary Information:**

The online version contains supplementary material available at 10.1186/s12866-022-02573-3.

## Introduction

*Bifidobacterium* is a genus of gram-positive, anaerobic microorganisms that are commonly found in the intestine of humans and animals [1, 2]. Some strains of *Bifidobacterium* have attracted significant attention due to their probiotic function in regulating microbiota and immune metabolism [3, 4]. *Bifidobacterium catenulatum* (*B. catenulatum*) is an important member of the genus; some of its strains demonstrate favourable probiotic characteristics, such as the preclinical treatment of acute liver injury [5], in vitro inhibition of pathogenic bacteria as well as the ability to stay alive in yoghurt for a long period [6]. These potential probiotic properties suggest that *B. catenulatum* may be a candidate for probiotics in food or medicine.

*Bifidobacterium* has long been considered an important intestinal symbiotic bacterium co-evolving with its hosts. In the previous studies, the dominant species of *Bifidobacterium* in the gut of infants and adults are usually different [1, 7, 8]. For example, *B. bifidum*, *B. longum* subsp. *infantis* and *B. breve* are commonly found in the gut of infants, while *B. adolescentis* and *B. catenulatum* usually appear in the intestinal tract of adults [9–12]. According to the latest taxonomy [13], *B. catenulatum* contains two subspecies, *B. catenulatum* subsp. *kashiwanohense* and *B. catenulatum* subsp. *catenulatum*. These two subspecies have different preferences in infant and adult intestine [14, 15]. *B. catenulatum* subsp. *catenulatum* is usually the dominant *Bifidobacterium* species in the adult gut [12, 15, 16]. Although *B. catenulatum* subsp. *catenulatum* is also present in infants, it is not the dominant *Bifidobacterium* species in the infant gut microbiota [15], and it is shown to be shared between in single mother-infant pairs [1]. *B. catenulatum* subsp. *kashiwanohense* is a greatly rare species, which lives mainly in the gut of infants [14]. Current research suggests that *B. catenulatum*’s adaptation to different hosts is partially due to the functional preference of different subspecies, such as carbohydrate metabolism [14]. However, there is limited genomic evidence corresponding to the different functional preferences of the two subspecies. Therefore, it is necessary to fill the gap in the genomic knowledge of the genetic divergence and functional differentiation of the two subspecies; the additional information will be useful for supplementing the existing knowledge on the bacterium and providing scientific support for their purported health benefits.

In-species comparative genomics analysis allows for a deeper understanding of the individual characteristics between genomes [17]. However, because the *Bifidobacterium* genus is strictly anaerobic, thus it is difficult to culture and easily contaminated by other species [18]. The number of published *B. catenulatum* genomes is currently limited. Recently, newly developed sequencing technologies have begun to uncover the *B. catenulatum* genomes [19]. While there have been genomic analyses of this species, most of the genomic information of *B. catenulatum* remains unexplored.

In the current study, a total of 19 genomes of *B. catenulatum* species were analysed, including 12 *B. catenulatum* subsp. *catenulatum* and 5 *B. catenulatum* subsp. *kashiwanohense* from the Refseq database, and 2 newly sequenced (IMAUFB085 and IMAUFB087) strains. The study dissected the genetic background and functional genomic information in *B. catenulatum* using comparative genomic approaches. This work not only provides general insights into the genomic differences between two subspecies of *B. catenulatum* but also reveals the key factors leading to their divergence.

## Results

### Average nucleotide identity (ANI) and Total nucleotide identity (TNI) analyses of *B. catenulatum* strains

**Fig. 1:**
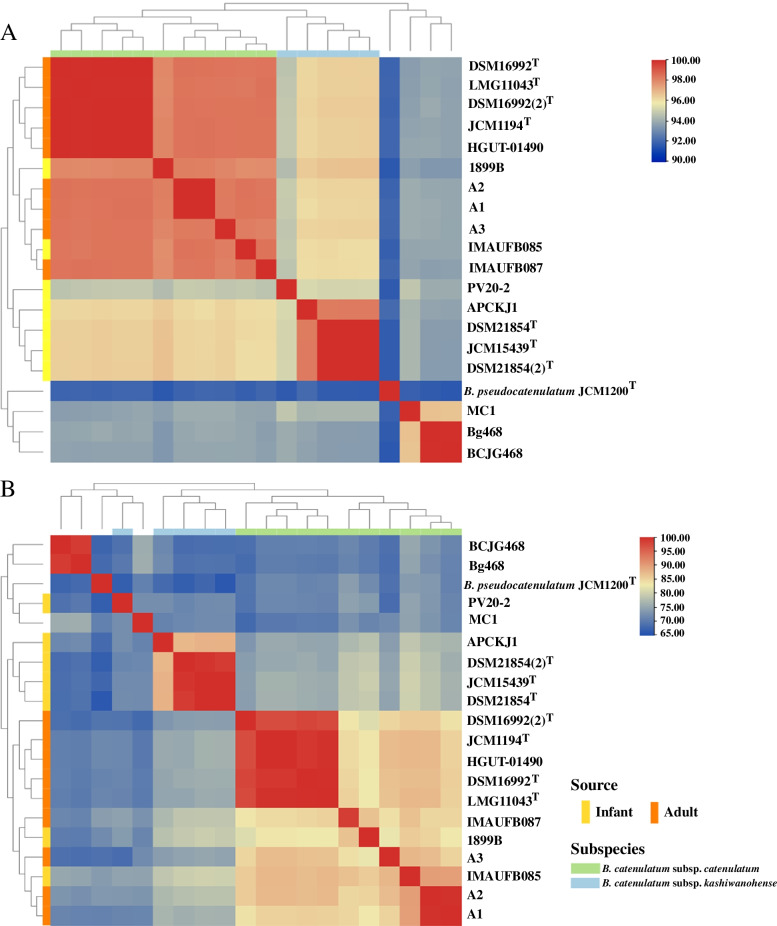
Heatmap of ANI (**A**) and TNI (**B**) based on the sequences of 20 genomes. The location and isolated resource of primary *B. catenulatum* isolates were annotated

The sequence similarity and taxonomic status among the strains used in this study were confirmed by calculating the pairwise ANI (Fig. 1A) and TNI (Fig. 1B) values of all 20 genome assemblies. Strains with an ANI value of over 95% are generally considered the same species [20]. The ANI and TNI analyses produced similar clustering results, displaying distinct subspecies branches. IMAUFB085 and IMAUFB087 were grouped with most of the *B. catenulatum* subsp. *catenulatum* strains; their ANI values compared to that of *B. catenulatum* subsp. *catenulatum* JCM1194^T^ were 98.41% and 98.42%, and TNI values were 87.45% and 84.48%, respectively. These results confirmed the classification of IMAUFB085 and IMAUFB087 as *B. catenulatum* subsp. *catenulatum*.

ANI analysis revealed that 3 *B. catenulatum* subsp. *catenulatum* strains, JGBg468, BCJG468 and MC1, significantly differed from the other *B. catenulatum* subsp. *catenulatum* strains; their ANI values compared to JCM1194^T^ were 93.83%, 93.88% and 93.86%, respectively, less than the threshold value of 95%. Therefore, these strains were subsequently excluded. In addition, cluster analysis distinguished two subspecies. The ANI value was greater than 95% between the 2 subspecies groups, and greater than 98% within the subspecies, indicating that these strains belonged to the same species.

### Comparison of general genomic features between two subspecies

The general information of the strains shows that all *B. catenulatum* subsp. *kashiwanohense* strains are derived from infants, while only two strains of *B. catenulatum* subsp. *catenulatum* are known to be infantile isolates (Table S1). The genomic features of 19 *B. catenulatum* genomes are summarised (Table 1) and the genomic characteristics within the *B. catenulatum* species exhibited different degrees of difference. The genome size and GC content of *B. catenulatum* isolates were 2.16 ± 0.13 Mb and 56.21 ± 0.11%, respectively. A comparison of the basic genomic characteristics of the two subspecies (Fig. S1) indicated that the genome size of *B. catenulatum* subsp. *kashiwanohense* (2.36 ± 0.05 Mb) was significantly larger than that of *B. catenulatum* subsp. *catenulatum* (2.09 ± 0.07 Mb) (*p* = 0.0021), while there were no significant differences in GC content (*p* > 0.05). The substantial genomic differences reflected the speciation boundaries of the two subspecies, while the similarity in GC content represented a close relationship between them [21, 22]. In addition, *B. catenulatum* subsp. *kashiwanohense* contained more coding genes (CDSs) than *B. catenulatum* subsp. *catenulatum* (*p* = 0.0046) and there were no statistical differences in the number of tRNAs (*p* > 0.05).

**Table 1:** General genomic features of *B. catenulatum* genomes

Collection strain	Genome size (Mb)	GC content (%)	No of CDSs	No of tRNAs
IMAUFB087	2.01	56.06	1,834	56
IMAUFB085	1.98	55.94	1,781	54
*B. catenulatum* subsp. *catenulatum* JCM1194^T^	2.08	56.20	1,616	56
*B. catenulatum* subsp. *catenulatum* DSM16992	2.06	56.10	1,606	56
*B. catenulatum* subsp. *catenulatum* LMG11043	2.08	56.11	1,515	56
*B. catenulatum* subsp. *catenulatum* DSM16992(2)	2.11	56.41	1,616	56
*B. catenulatum* subsp. *catenulatum* 1899B	2.12	56.25	1,656	56
*B. catenulatum* subsp. *catenulatum* A2	2.02	56.15	1,584	54
*B. catenulatum* subsp. *catenulatum* A1	2.06	56.21	1,659	56
*B. catenulatum* subsp. *catenulatum* A3	2.15	56.36	1,707	59
*B. catenulatum* subsp. *catenulatum* HGUT-01490	2.08	56.20	1,615	56
*B. catenulatum* subsp. *kashiwanohense* PV20-2	2.37	56.12	1,876	58
*B. catenulatum* subsp. *kashiwanohense* JCM15439^T^	2.34	56.30	1,842	54
*B. catenulatum* subsp. *kashiwanohense* APCKJ1	2.45	56.20	1,968	54
*B. catenulatum* subsp. *kashiwanohense* DSM21854	2.31	56.20	1,758	53
*B. catenulatum* subsp. *kashiwanohense* DSM21854(2)	2.32	56.30	1,854	68

The overall genomic differences between the two subspecies were further explored using the BLAST Ring Image Generator (BRIG) to graphically compare *B. catenulatum* strains with *B. catenulatum* subsp. *kashiwanohense* strain JCM15439^T^ as the reference (Fig. S2). Overall, most of the sequences in JCM15439^T^ were also in all other strains, and the genomes were more than 90% identical. However, two large genomic gaps (GGs) existed separately in the two newly sequenced strains, IMAUFB085 and IMAUFB087, which had less than 70% of the matched degree compared to JCM15439^T^. In general, the GG sequences represent hypothetical CDSs, genomic islands or prophages [23]. These data indicate that these two strains have many unknown genomic information to be explored.

### Phylogenetic divergence of two subspecies of *B. catenulatum*

**Fig. 2:**
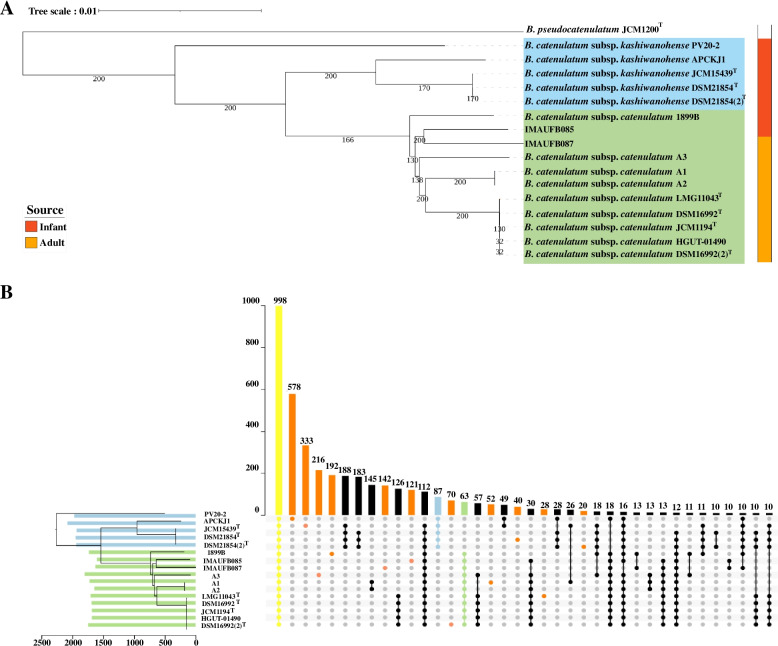
Phylogenetic tree of two subspecies of *B. catenulatum.* Phylogenetic NJtree of *B. catenulatum* species and taken *B. pseudocatenulatum* JCM1200^T^ as the outgroup. Bootstrap was set as 1000. All the *B. catenulatum* strains were annotated to isolate location and source. The scale bars represent 0.01 substitutions per site (**A**). UpSet diagram showing shared and unique core genes distribution among *B. catenulatum* strains. The horizontal bars represent the total number of genes identified of individual strains. The vertical bars or intersections represent the number of genes that were regulated by one or more strains. The orange dots represent unique genes and the yellow dots represent core genes. The green items represent information about *B. catenulatum* subsp. *catenulatum*, and the blue items represent information about *B. catenulatum* subsp. *kashiwanohense*. Groups with fewer than 10 genes were filtered (**B**)

Classification of species and establishment of intra-specific relationships are frequently based on phylogenetic analysis. A phylogenetic tree based on 785 core genes was constructed that confirmed the subspecies divergence of *B. catenulatum* (Fig. 2A). 16 *B. catenulatum* strains were clearly divided into two subspecies, indicating the genetic differences between the two subspecies at the genomic level. Interestingly, the annotation of the source of the isolates suggested a significant cluster. Infant isolates, including all *B. catenulatum* subsp. *kashiwanohense* strains and 2 *B. catenulatum* subsp. *catenulatum* strains, exhibited intra-specific genetic similarity, while the rest were adult isolates in another cluster, indicating close phylogenetic relationships. These data suggest that the divergence of the *B. catenulatum* strains likely dependent on their hosts. *B. catenulatum* may adapt its functions to infant and adult intestines respectively, thus gradually differentiating into different subspecies.

### Constructing the pan-core genome of *B. catenulatum*

The gene pool of a population contains all the genetic material and functions of a species. Roary was used to calculate the pan-core genome of the 16 *B. catenulatum* strains; a total of 4608 pan genes were searched. The genetic distribution of *B. catenulatum* showed that the two subspecies of *B. catenulatum* shared 998 core genes (21.66%) (Fig. 2B). There were unique core gene sets in the 2 subspecies, with 87 unique core genes in *B. catenulatum* subsp. *kashiwanohense* and 63 in *B. catenulatum* subsp. *catenulatum* (Table S2). The unique core gene sets of two subspecies are involved in the metabolism of diversity functions, such as carbohydrate (group_1783, group_2168, et al.), amino acid (*met*I, group_2203, et al.), protein (group_1013, group_1298, et al.), and so on. These unique core genes may play a role in the differentiation of their species [2, 22, 24], although some are hypothetical proteins. Additionally, there were different numbers of strain-specific genes in the *B. catenulatum* subspecies; their numbers ranged from 20 to 578, suggesting the potential genetic diversity among *B. catenulatum* species.

Subsequently, the pan-core gene curves for the genomes of the *B. catenulatum* species were established (Fig. S3A). With the addition of the new genomes, the number of pan genes increased, indicating the existence of an open pan-genome within the species of *B. catenulatum*. In contrast, the number of core genes was not expected to be significantly reduced by the addition of the new genomes since the exponential trendline reached the number of 1000. Notably, *B. catenulatum* subsp. *catenulatum* has a fairly open pan-core genome (Fig. S3B), while *B. catenulatum* subsp. *kashiwanohense*’s genome tends to be closed (Fig. S3C). These results indicate that *B. catenulatum* subsp. *catenulatum* may have flexible environmental adaptability, while *B. catenulatum* subsp. *kashiwanohense* exists in a more specific and conserved habitat [25]. However, due to the limitation of *B. catenulatum* genome number, this deduction needs more sequencing results to confirm.

### Comparison of the main functions between two subspecies

**Fig. 3:**
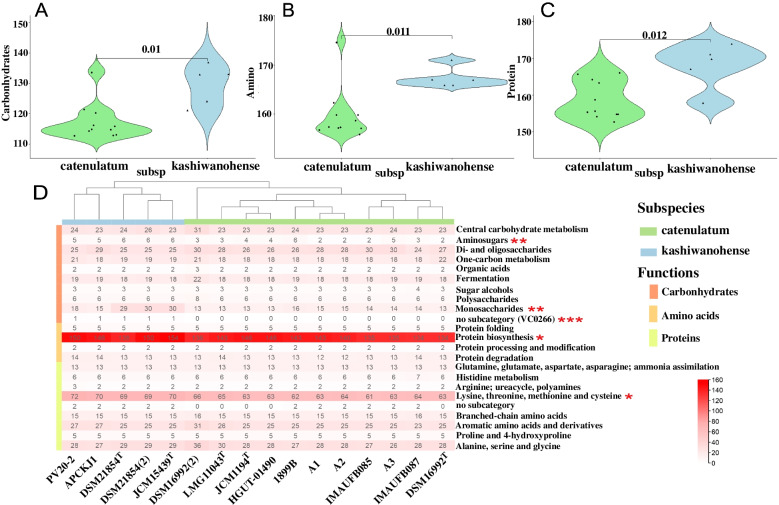
Comparison of the main functions between *B. catenulatum* subsp. *catenulatum* and *B. catenulatum* subsp. *Kashiwanohense.* Significant difference of genes in major functions. Amino acid derivatives (**A**); Protein metabolism (**B**); Carbohydrate metabolism (**C**). Heat maps of detailed subcategories of main functions (**D**). The numbers in the heat map indicate the number of copies of functional genes. *p < 0.05, **p < 0.01, ***p < 0.001

The above results have uncovered the genetic differences between the two subspecies at the general genomics level, which are usually associated with functional differentiation [24]. Therefore, it is necessary to conduct further functional genomic comparisons between the two subspecies of *B. catenulatum*. Their functional genomic differences were obtained by annotating all the strains through the RAST website. The functional annotations of 16 *B. catenulatum* genomes were examined in 23 functional categories (Fig. S4). These results suggest that the function of amino acid derivatives (21.06%) is the most highly represented category within *B. catenulatum* followed by protein metabolism (21.00%) and carbohydrate metabolism (15.73%) (Fig. S4). It indicates that the three functions are the main ability to utilise substrates by *B. catenulatum*. The comparison of the main functional differences between the two subspecies showed the subspecies differ significantly in their metabolism of carbohydrates (*p* = 0.01), amino acids (*p* = 0.011) and proteins (*p* = 0.012) (Fig. 3A, 3B, 3C).

In view of the remarkably significant differences in the metabolic functions of carbohydrates, amino acids and proteins in the two subspecies, the detailed categories of the main functions were compared in the two subspecies (Fig. 3D). It showed that the two subspecies are divided into two clusters, and the difference in functional genes was most significant in carbohydrates, it mainly lie in aminosugars, monosaccharides and an unclassed subcategory related to carbohydrates. In addition, the most significant differences in protein occurred in genes related to protein biosynthesis, and for amino acids it occurred in functional genes of lysine, threonine, methionine, and cysteine. This suggests that the functional difference in these significant subcategories is the key to the difference in the functional genomes of the two subspecies of *B. catenulatum*. Because of the most significant difference between the two subspecies was in carbohydrate function, the *B. catenulatum* genes involved in carbohydrate utilisation were analysed.

### Different carbohydrate utilisation patterns in two subspecies of *B. catenulatum*

**Fig. 4:**
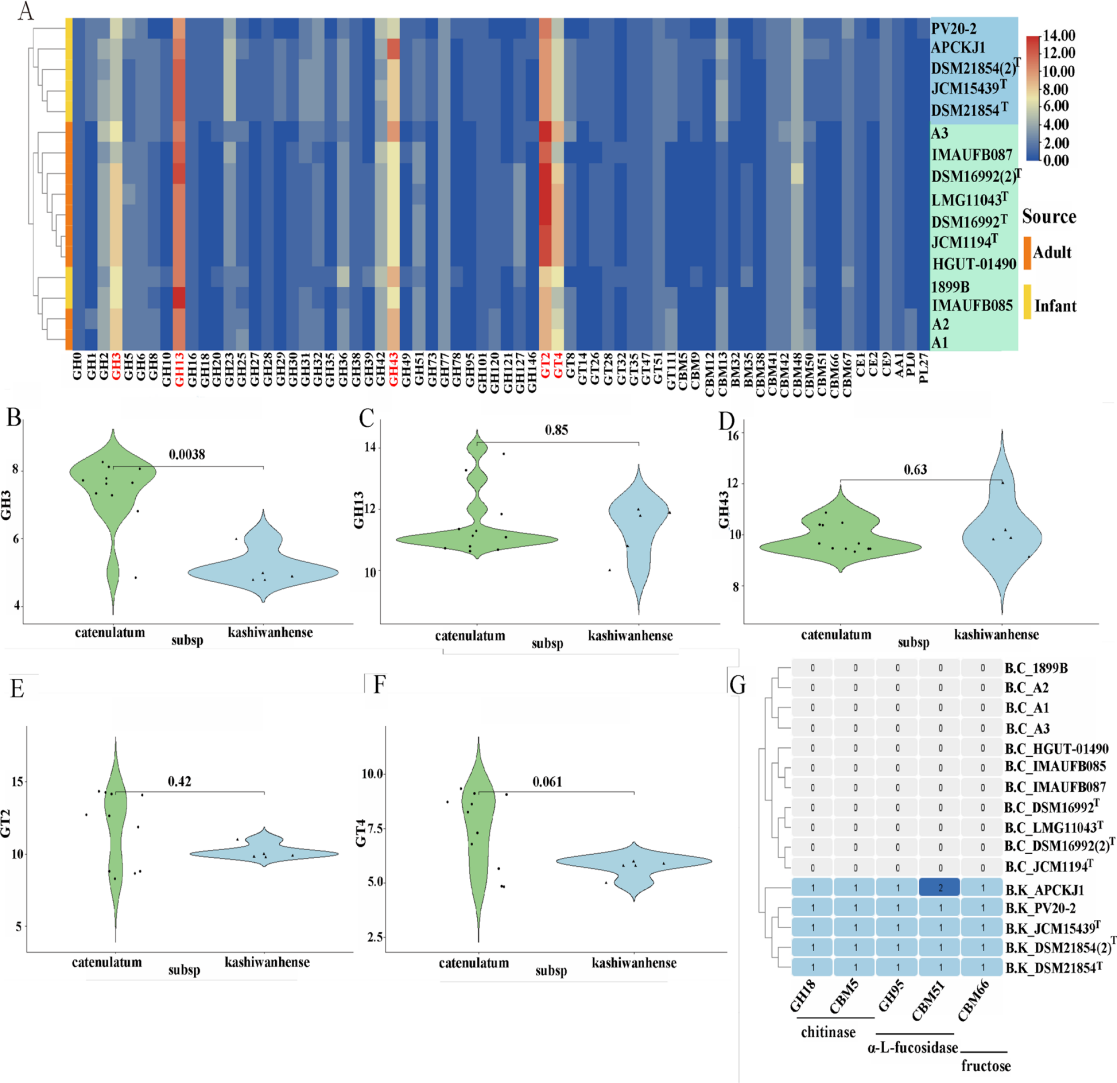
Prediction of CAZymes in 16 *B. catenulatum* strains. The Heatmap of CAZymes in *B. catenulatum*. The isolated source of strains was annotated (**A**). The significance analysis of the key CAZymes families between two subspecies of *B. catenulatum* including GH3 (**B**), GH13 (**C**), GH43 (**D**), GT2 (E), and GT4 (**F**). Specific CAZymes in *B. catenulatum* subsp. *kashiwanohense* (G)

The carbohydrate utilisation abilities of *B. catenulatum* subspecies at the genomic level were compared by analysing the functional genes of carbohydrate-active enzymes (CAZymes) of 16 *B. catenulatum* strains. As shown in Fig. 4A, 16 *B. catenulatum* strains were distributed in all six carbohydrate-active enzyme families, indicating that they had rich carbohydrate functions. Notably, the clustering results of CAZymes were roughly consistent with those of the phylogenetic trees in that the two subspecies were distinct. This finding not only suggests that the two subspecies have different metabolic patterns in terms of carbohydrate utilisation, but also indicates that CAZymes-related genes are closely associated with the divergence of *B. catenulatum* subspecies.

Among the identified GH families in *B. catenulatum* species, the most dominant ones were GH3, GH13 and GH43; meanwhile, GT2 and GT4 were the main carbohydrate enzyme families within *B. catenulatum* species. Comparing the main carbohydrate hydrolase families in the subspecies revealed the number of GH3 family members was significantly higher in *B. catenulatum* subsp. *catenulatum* than those in *B. catenulatum* subsp. *kashiwanohense* (*p* = 0.0038, Fig. 4B). GH3 is mainly involved in the metabolism of plant-derived glycan common in the adult diet, such as β-glucosidase and xylosidase [26]. However, there was no statistically significant difference in the function of GH13, GH43, GT2 and GT4 between the two subspecies (*p* > 0.05) (Fig. 4C, 4D, 4E, 4F). Therefore, GH3 may be a key factor in the divergence of carbohydrate functional genes between the two subspecies of *B. catenulatum*.⁠

Analysis of the specific CAZymes of *B. catenulatum* subsp. *kashiwanohense* revealed five families that only existed in the subspecies, including GH18, CBM5, GH95, CBM51 and CBM66 (Fig. 4G). The CBM family is primarily responsible for banding carbohydrates. In addition, the GH18 family often combines with CBM5 to participate in the function of chitinase, and CBM66 mainly assists in the degradation of fructose [27]. In particular, the GH95 family is specifically involved in the production of α-L-fucosidase, the most abundant substance in HMO and closely related to the function of infant-specific species [28]. Additionally, the CBM51 family helps GH95 enzymes pick up fucose to metabolise HMO [29]. These CAZyme families CBM51 and GH95 may be conducive to the colonisation of *B. catenulatum* subsp. *kashiwanohense* in the intestines of infants, especially the utilization of HMO, in contrast to the abundance of plant-derived glycan of *B. catenulatum* subsp. *catenulatum*, further suggesting the bias of the two subspecies in carbohydrate utilisation. In addition, GH29 enzymes often interact with GH95 enzymes to utilise HMO [30], and the study found that GH29 is only in *B. catenulatum* subsp. *kashiwanohense* except for PV20-2.

### Identification of HMO gene clusters in *B. catenulatum* genomes

**Fig.5:**
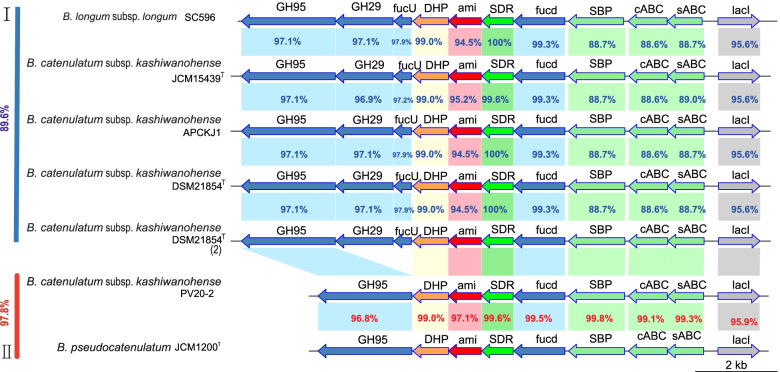
HMO gene clusters in *B. catenulatum* subsp. *kashiwanohense* and two reference clusters in *Bifidobacterium*. Arrows represent genes, and numbers on top of each gene indicate the locus tag number in the respective genome. Numbers inside the arrows indicate percent identity between corresponding genes and homologs relative to reference. The numbers outside on the left indicate percent identity of full clusters relative to reference. SBP: Solute Binding Protein; cABC: carbohydrate ABC transporter; sABC: sugar ABC transporter; SDR: SDR family oxidoreductase; DHP: dihydrodipicolinate synthase family protein; *fuc*U: L-fucose mutarotase; *fuc*d: fuconate dehydratase

Considering the specific utilisation of fucosylated HMO (FHMO) by GH29 and GH95 enzymes, the FHMO gene cluster in *B. catenulatum* were subsequently examined. Two *Bifidobacterium* strains (*B. longum* subsp. *longum* SC596 and *B. pseudocatenulatum* JCM1200^T^) with typically structural FHMO gene clusters were selected as the reference [31] for the search for the homologous FHMO gene cluster in all of the *B. catenulatum* genomes. The homologous alignment showed an integrated FHMO gene cluster in all *B. catenulatum* subsp. *kashiwanohense* genomes but not in *B. catenulatum* subsp. *catenulatum* (Fig. 5), further confirming the unique ability to utilise HMO by *B. catenulatum* subsp. *kashiwanohense*. In the study, two different structures of FHMO gene clusters, named type I and type, ⁠were found in *B. catenulatum* subsp. *kashiwanohense* (Table S3). Type I shared 89.6% homology with *B. longum* subsp. *longum* SC596. The size of type I was about 13.0 kb, including 11 open reading frames (ORF), manifested as GH95, GH29, *fuc*U, dihydrodipicolinate synthase family protein (DHP), amidohydrolase family protein, SDR family oxidoreductase, fuconate dehydratase, three ABC transporters and *lac*I. Meanwhile, type II shared 97.8% homology with *B. pseudocatenulatum* JCM1200^T^; it was only found in PV20-2 and lacked GH29 and *fuc*U genes, consistent with the results of CAZymes.

Notably, the GC content of the FHMO gene clusters in *B. catenulatum* subsp. *kashiwanohense* was significantly lower than the entire subspecies (Fig. S5), suggesting that its FHMO gene clusters might be obtained through horizontal gene transfer (HGT) [32–34]. The identification of the FHMO gene clusters in *B. catenulatum* subsp. *kashiwanohense* further confirmed its advantage of HMO utilisation, thus providing genomic evidence for its adaptability in the infant intestine.

## Discussion

As a typical intestinal symbiotic bacteria, *Bifidobacterium* has experienced a long and extensive evolutionary process in human hosts [1]. For example, *B. catenulatum* has evolved into two subspecies, *B. catenulatum* subsp. *kashiwanohense* and *B. catenulatum* subsp. *catenulatum*. Previous studies have revealed that *B. catenulatum* subsp. *kashiwanohense* and *B. catenulatum* subsp. *catenulatum* have a close phylogenetic relationship [2]. Here, phylogenetic reconstruction has revealed genetic differences between the two subspecies. The genome size and the number of the CDSs of *B. catenulatum* subsp. *catenulatum* were significantly lower than that of *B. catenulatum* subsp. *kashiwanohense*. Also, both subspecies have a unique core gene set, such results represent a marker of genetic divergence [22]. In addition, there was obvious host differentiation in *B. catenulatum*, that *B. catenulatum* subsp. *catenulatum* is more present in adult intestines [1, 2], while *B. catenulatum* subsp. *kashiwanohense* commonly be confirmed as an infant-associated species [14]. Although *B. catenulatum* subsp. *kashiwanohense* is only rarely reported, previous studies have shown that *kashiwanohense* can be present in breast milk samples [15] and utilize milk-derived substrates, suggesting that the infant gut may be its main niche [14]. In this study, the possible association between subspecies divergence and the host was further explored through functional genomic comparisons to explain the divergence of *B. catenulatum* at the genomic level.

*Bifidobacterium* is a genus of saccharolytic microorganisms whose ability to utilise indigestible carbohydrates is essential for their establishment in the gastrointestinal tract [35]. In this study, functional genomics revealed significant differences in the carbohydrates consumed by the subspecies of *B. catenulatum*. Notably, the CAZymes cluster results are consistent with the phylogenetic tree analysis, suggesting that the functional differences in carbohydrates may be related to the genetic divergence of *B. catenulatum*. This study found that the GH3 content of *B. catenulatum* subsp. *catenulatum* was significantly higher than that of *B. catenulatum* subsp. *kashiwanohense*. Previous studies have shown that GH3 is a key family in the evolution of *Bifidobacterium* and is involved in the degradation of plant polysaccharides [36]. The results here indicate that GH3 is also a key factor for the divergence of *B. catenulatum* in carbohydrate function*.* Studies have shown that the gut environment in adults is more complex than in infants because adults typically consume more difficult-to-digest carbon sources, such as plant-based dietary fibre [9, 10]. Kim et al. found that *B. catenulatum* strains can degrade fructooligosaccharides (FOS) in nutritionally restricted environments [37]. Previous studies have shown that a low-fiber diet in adults can cause a significant increase in the abundance of *B. catenulatum* [38]. Here, the results demonstrate that *B. catenulatum* subsp. *catenulatum* has more GH3 that utilises plant-derived glycans; therefore, the subspecies is conducive to the decomposition of difficult-to-use plant-derived glycans in the adult gut.

On the other hand, infants, especially those who are breastfed, have many HMOs in their intestines. HMO is a prebiotic unique to breast milk and is especially enriched in human breast milk [39]. The ability of infant-specific *Bifidobacterium* to metabolise HMO has been recognised as a specific marker of its adaptive colonisation and beneficial for strengthening the immune system in infants [40]. For *B. catenulatum* subsp. *kashiwanohense*, which is characterised by infant adaptation [14], its two specific CAZymes, namely GH95 and CBM51, which are notable. GH95 mainly utilises fucosyllactose, a major component of HMO [41]. On the other hand, CBM51 is beneficial to GH95 and helps it pick up FHMO [29]. Thus, this study suggests that GH95 and CBM51 act synergistically in the utilisation of FHMO by *B. catenulatum* subsp. *kashiwanohense*. In particular, GH29 is often identified with GH95 as the family of metabolic HMO [30]. In *B. catenulatum* subsp. *kashiwanohense*, all strains except PV20-2 contain GH29. Therefore, the study suggests that these three families (GH29, GH95 and CBM51) play an important role in the colonisation of *B. catenulatum* subsp. *kashiwanohense* in the infant intestine.

Based on the findings related to the HMO-related families, this study further confirms the existence of relatively conserved HMO gene clusters in *B. catenulatum* subsp. *kashiwanohense* while not in *B. catenulatum* subsp. *catenulatum*. These HMO gene clusters are highly homologous to those in other typical infantile adapted *Bifidobacterium* that are connected to the GH95 and GH29 families. Only the PV20-2 strain lacks GH29 and *fuc*U, while the genome of PV20-2 shares high homology with the HMO gene cluster of *B. pseudocatenulatum* JCM1200^T^, which can grow in purified FHMO [42], the lack of these two genes appears to have little effect on the overall ability to use FHMO. Given that the reference genomes in HMO gene clusters are all from infants, their clusters have been demonstrated to be conducive to their utilisation of HMO [35, 42]. This study suggests that *B. catenulatum* subsp. *kashiwanohense* may have a similar utilisation mechanism of FHMO for adaptive survival in the infant intestine [30, 35, 41]. Previous studies [14, 43] had confirmed through gene expression experiments that the fucosyllactose transporters in *B. catenulatum* subsp. *kashiwanohense* JCM15439^T^ and HMO genes in *B. catenulatum* subsp. *kashiwanohense* APCKJ1 endowed them with ability of HMO consumption, thus contributing to their adaptation in the HMO-rich environments. Given the high similarity of the HMO gene clusters in *B. catenulatum* subsp. *kashiwanohense*, this ability to consume HMO may be an intrinsic characteristic of this subspecies. In addition, a group_2168 gene codes L-fuconate dehydratase specifically exists in the unique set of *B. catenulatum* subsp. *kashiwanohense* (Table S2), which would be one of the markers of genetic divergence [22] and is consistent with the conclusion that it adapts to metabolizing FHMO. Notably, *B. catenulatum* subsp. *catenulatum* 1899B and IMAUFB085 belong to infant isolates, but no HMO genes were found in them, further confirming that possession of HMO genes is a genetic trait of *B. catenulatum* subsp. *kashiwanohense*.

The complex carbohydrate environment in the human gut can drive HGT events in *Bifidobacterium*, and commonly occurs between closely related species [44, 45]. Garrido et al. [11] propose that the HMO gene clusters have transferred from *B. longum* subsp. *infantis* to *B. longum* subsp. *longum* during evolution. Notably, the HMO gene cluster in *B. catenulatum* subsp. *kashiwanohense* in this study showed a significant decrease in GC content. Previous reports confirmed that HGT fragments differ from native genes in GC content [33, 34]. Thus we infer that the HMO clusters of *B. catenulatum* subsp. *kashiwanohense* were obtained by HGT, which were important in the genomic evolution of species [11]. At present, these types of HMO gene clusters have been found in typical infant-derived strains, such as *B. breve*, *B. longum* and *B. pseudocatenulatum* species, and they have high homology with each other [30, 35, 42]. This study proposes that *B. catenulatum* subsp. *kashiwanohense* acquired HMO gene clusters through HGT from other proximal species (such as *B. longum*), the acquisition of HGT contributed to the specific function of genome divergence and HMO utilisation.

Although the two subspecies of *B. catenulatum* are closely phylogenetically related and share a common ancestor [2], previous studies have confirmed that they showed different tendencies adapted in infants and adult intestines [9, 10, 14]. Taken together, given that the carbohydrate genetic pattern of the two subspecies was consistent with the phylogenetic relationship, we speculated that the *B. catenulatum* species evolved to retain the competitive carbohydrate function genes to adapt to the intestinal environment of infants and adults respectively, driving the emergence of two subspecies. Our results are similar to the divergence of *B. longum*, for the *infantis* subspecies of it has specific genes related to the metabolism of HMO and is more suitable for breast-feeding infant intestines, while the *longum* subspecies is present in both infant and adult hosts but has more genes for the utilization of plant-derived glycan and is more suitable for adult diets [35]. The example of this divergence of species in different hosts seems to suggest a potential pattern of genetic divergence of *Bifidobacterium*, in which infant and adult wealthy species have more HMO genes and plant-derived glycan genes respectively in the human gut in order to adapt to their respective hosts.

## Conclusions

In summary, this study proposes that the *B. catenulatum* species evolved to retain the competitive carbohydrate function genes to adapt to the respective intestinal environment in infants and adults, driving the emergence of two subspecies. This study has provided genomic evidence for the potential host adaptation phenomenon of *B. catenulatum* in infant and adult intestines. However, the number of *B. catenulatum* strains is limited; more strains will need to be sequenced in the future to dissect further the mechanism underlying their genetic divergence.

## Methods

### Bacterial strains, DNA extraction and publicly available assemblies

The two *B. catenulatum* strains (IMAUFB085 and IMAUFB087) that sequenced in this study were provided by the Lactic Acid Bacteria Collection Center (LABCC). Moreover, IMAUFB085 was isolated from infant faeces and IMAUFB087 from adult faeces in Tibet, China [46].

The two strains were cultured under anaerobic conditions in the Man Rogosa and Sharpe (MRS) broth with L-cysteine hydrochloride at 37 °C for 24 h. DNA extraction was performed using the TIANamp Bacteria DNA Kit. Genomic DNA was quantified using a TBS-380 fluorometer. High-quality DNA samples were obtained to construct fragment libraries.

In addition, other 17 *B. catenulatum* genomes were obtained from the National Coalition Building Institute (NCBI, https://www.ncbi.nlm.nih.gov/) on 4 February 2021, including that of type strains, namely *B. catenulatum* subsp. *catenulatum* (JCM1194^T^) and *B. catenulatum* subsp. *kashiwanohense* (JCM15439^T^) (Table S1). Additionally, the *B. pseudocatenulatum* strain (JCM1200^T^) in the *B. adolescentis* group, most closely related to *B. catenulatum* according to the phylogenetic relationship of *Bifidobacterium* genus in a previous study [2], were downloaded to infer phylogenetic relationships across species within it.

### Genome sequencing and assembly

Genome sequencing was performed using the Illumina HiSeq platform to generate 150-bp paired-end reads for each sample. Then, the sequences were filtered through the Illumina HiSeq system. The high-quality sequences were assembled using SOAPdenovo2 [47] on a 64-bit Linux system. High-quality data corresponding to a sequencing depth of about 387-fold, was generated for each strain. In addition, local inner gaps were filled, and single-base errors were corrected using GapCloser (http://sourceforge.net/projects/soapdenovo2/files/GapCloser/).

### Genome annotation

In this study, all the general genomic information of *B. catenulatum* genomes was generated using self-made Perl scripts with statFASTA.pl. The functional gene information of *B. catenulatum* was obtained by performing the gene prediction and preliminary annotation of all *B. catenulatum* genomes through the Rapid Annotation using Subsystems Technology (RAST) server (https://rast.nmpdr.org/rast.cgi). In addition, tRNA genes were identified using tRNAscan-SE (http://trna.ucsc.edu/tRNAscan-SE/).

### ANI and TNI

The genetic relatedness between the two *B. catenulatum* subspecies was evaluated, and the taxonomic status of the strains in this study was confirmed by analysing the ANI and TNI values of all the strains. *B. pseudocatenulatum* JCM1200^T^, the type strain most phylogenetically related to *B. catenulatum* [2], was included in the comparison. All pairwise ANI values were calculated according to the method proposed by Goris et al. [48]. TNI values were calculated according to the method proposed by Chen et al. [49]. Finally, the clustering heat map was drawn using TBtools [50].

### Construction of pan-core genome and strain-specific genes

The annotated genomes of *B. catenulatum* were obtained using Prokka v1.12 [51] and processed using Roary v3.8.0 [52] to identify the pan genes, core genes and specific genes using the default parameters. The intersection groups, representing the unique sets of genes identified only between the intersected genomes, were visualised using the UpSet diagram in TBtools [50].

### Phylogenetic analysis

The core gene alignment from Roary was used in TreeBeST [53] with 1,000 bootstrap iterations to build a phylogenetic NJtree through Neighbor-Joining (NJ) [54]. The phylogenetic trees were then visualised and annotated using iTOL (https://itol.embl.de/).

### BRIG (BLAST Ring Image Generator)

BRIG v0.95 [55] was adapted to compare the genomes of *B. catenulatum* strains based on a JAVA language environment. All settings use default parameters. The image of the circular genomes was also generated through BRIG.

### CAZymes online annotation

The identification of CAZymes across the *B. catenulatum* genomes was carried out using the dbCAN2 meta server (http://bcb.unl.edu/dbCAN2/), using three annotation tools, including HMMER, DIAMOND and Hotpep searches [56]. The database includes glycosyltransferases (GTs), glycoside hydrolases (GHs), carbohydrate esterases (CEs), polysaccharide lyases (PLs), auxiliary activity (AA) and carbohydrate-binding modules (CBMs). According to the annotation results, the detailed information on the active carbohydrate enzyme family was checked on the CAZyme website (http: //www.aczy.org/).

### Detection of the HMO gene clusters

Taking *B. longum* subsp. *longum* SC596 and *B. pseudocatenulatum* JCM1200^T^ as the reference, which possess typical HMO gene clusters. In addition, the genome of SC596 was obtained from the IMG database [57]. The corresponding protein-encoding sequences were extracted from the genomes and compared using BLASTp with default parameters from the NCBI website. The cut-off values of 50% of similarity across 50% of protein length and a 0.0001 e-value as a significance for the identification of homologous proteins. The recognised HMO gene clusters were visualised using the genoplotR package.

### Statistical analysis

The data were presented as means ± SEM. The Wilcoxon signed-rank test was used to verify the significance of the difference between the groups, and visualisation was performed using the ggpubr packages in R (4.0.3). Lastly, significance was set at a *p*-value of less than 0.05.

### Data availability

The assembly and Sequence Read Archive (SRA) data of the two newly isolated sequences in this work were submitted as a Whole Genome project (BioProject No. PRJNA751426) at GenBank under the accessions JAIEWL000000000 (IMAUFB087) and JAIEWM000000000 (IMAUFB085) (available at https://www.ncbi.nlm.nih.gov/bioproject/PRJNA751426). The phylogenetic trees and alignment files in this study were submitted to the TreeBASE web (Accession No. 28852) (available at http://purl.org/phylo/treebase/phylows/study/TB2:S28852).

## Supplementary Information

**Additional file 1: TableS1.** General information of *B. catenulatum* genomes.

**Additional file 2: Table S2.** Unique core genes oftwo subspecies of *B. catenulatum*.

**Additional file 3: Table S3. **Information on HMOgene clusters of *B. catenulatum *subsp. *kashiwanohense*and its references.

**Additional file 4: Fig. S1.** Comparison ofgenomic features between two subspecies of *B. catenulatum*, includinggenome size (A), GC Content (B), CDSs (C) and tRNA (D).

**Additional file 5: Fig. S2.** A display of thegenome circle map of 16 *B. catenulatum* genomes. The figure was generatedbased on comparison of 16 *B. catenulatum* genomes according to percentageidentity (100%, 90%, or 70%). The numbers on the rings from inside to outside,1: JCM15439^T^, 2: DSM21854(2)^T^, 3: DSM21854^T^,4: APCKJ1, 5: PV20-2, 6: HGUT-01490, 7: LMG11043^T^, 8: A1, 9: A3, 10:A2, 11: 1899B, 12: DSM16992^T^, 13: DSM16992(2)^T^, 14:JCM1164^T^, 15: IMAUFB085, 16: IMAUFB087.

**Additional file 6: Fig. S3.** Tendency curves forpan-core genomes of *B. catenulatum*. *B. catenulatum* (**A**), *B.catenulatum* subsp. *catenulatum* (**B**), *B. catenulatum*subsp. *kashiwanohense* (**C**).

**Additional file 7: Fig. S4.** Comparison of 23functional categories between *B. catenulatum* genomes. The numbers in theheat map indicate the number of copies of functional genes.

**Additional file 8: Fig. S5. **Comparisonof GC content between full-length genomes and FHMO clusters in *B. catenulatum*subsp. *kashiwanohense*.

## Data Availability

The assembly and Sequence Read Archive (SRA) data of the two newly isolated sequences in this work were submitted as a Whole Genome project (BioProject No. PRJNA751426) at GenBank under the accessions JAIEWL000000000 (IMAUFB087) and JAIEWM000000000 (IMAUFB085) (available at https://www.ncbi.nlm.nih.gov/bioproject/PRJNA751426). The phylogenetic trees and alignment files in this study were submitted to the TreeBASE web (Accession No. 28852) (available at http://purl.org/phylo/treebase/phylows/study/TB2:S28852).

## Abbreviations

LABCC: Lactic Acid Bacteria Collection Center
MRS: Man Rogosa and Sharpe
ANI: Average Nucleotide Identity
TNI: Total Nucleotide Identity
CDSs: Coding Sequences
GGs: Genome Gaps
NJ: Neighbor-Joining
RAST: Rapid Annotation using Subsystems Technology
BRIG: BLAST Ring Image Generator
HMOs: Human Milk Oligosaccharides
FHMO: Fucosylated HMO
*B. catenulatum*: *Bifidobacterium catenulatum*
CAZymes: Carbohydrate-active enzymes
GTs: Glycosyltransferases
GHs: Glycoside hydrolases
CEs: Carbohydrate esterases
PLs: Polysaccharide Lyases
AA: Auxiliary Activity
CBMs: Carbohydrate-Binding Modules
HGT: Horizontal Gene Transfer
FOS: Fructooligosaccharides
