# Supplementary material for: Comparative genomic analysis revealed genetic divergence between Bifidobacterium catenulatum subspecies present in infant versus adult guts
Source: BMC Microbiol. 2022 Jun 16;22:158. doi: 10.1186/s12866-022-02573-3 (PMC9202165; doi:10.1186/s12866-022-02573-3)
Supplement: Supplementary file 1 — Additional file 1: TableS1. General information of B. catenulatum genomes. [file 12866_2022_2573_MOESM1_ESM.docx]

**Table** **S1** General information of *B. catenulatum* genomes

| Collection strain | Isolation Source | Accession NCBI | No of  scaffolds | Reference |
| --- | --- | --- | --- | --- |
| IMAUFB087 | Adult feces | JAIEWL000000000 | 36 | [45] |
| IMAUFB085 | Infant feces | JAIEWM000000000 | 53 | [45] |
| *B. catenulatum* subsp. *catenulatum*  JCM1194^T^ | Adult feces | GCF_001025195.1 | 1 | [57] |
| *B. catenulatum* subsp. *catenulatum*  DSM16992^T^ | Adult feces | GCF_000173455.1 | 31 | [57] |
| *B. catenulatum* subsp. *catenulatum*  LMG11043^T^ | Adult feces | GCF_000741565.1 | 11 | [57] |
| *B. catenulatum* subsp. *catenulatum*  DSM16992(2)^T^ | Adult feces | GCF_000771025.1 | 164 | [57] |
| *B. catenulatum* subsp. *catenulatum*  1899B | Infant feces | GCF_002075855.1 | 14 | NCBI |
| *B. catenulatum* subsp. *catenulatum*  A2 | Adult feces | GCF_009160765.1 | 18 | [58] |
| *B. catenulatum* subsp. *catenulatum*  A1 | Adult feces | GCF_009160805.1 | 18 | [58] |
| *B. catenulatum* subsp. *catenulatum*  A3 | Adult feces | GCF_015548935.1 | 16 | [58] |
| *B. catenulatum* subsp. *catenulatum*  MC1 | Human feces | GCF_901212515.1 | 28 | NCBI |
| *B. catenulatum* subsp. *catenulatum*  BCJG468 | Human feces | GCF_902167655.1 | 36 | NCBI |
| *B. catenulatum* subsp. *catenulatum*  JG_Bg468 | Human feces | GCF_902167905.1 | 75 | NCBI |
| *B. catenulatum* subsp. *catenulatum*  HGUT-01490 | Adult feces | GCF_902381755.1 | 1 | [59] |
| *B.* *catenulatum* subsp. *kashiwanohense* PV20-2 | Infant feces | GCF_000800455.1 | 1 | [60] |
| *B.* *catenulatum* subsp. *kashiwanohense*  JCM15439^T^ | Infant feces | GCF_001042615.1 | 1 | [18] |
| *B.* *catenulatum* subsp. *kashiwanohense*  APCKJ1 | Infant feces | GCF_009684555.1 | 1 | [14] |
| *B.* *catenulatum* subsp. *kashiwanohense*  DSM21854^T^ | Infant feces | GCF_000741605.1 | 30 | [18] |
| *B.* *catenulatum* subsp. *kashiwanohense*  DSM21854(2)^T^ | Infant feces | GCF_000771545.1 | 68 | [18] |
