# Supplementary material for: Comparative genomic analysis revealed genetic divergence between Bifidobacterium catenulatum subspecies present in infant versus adult guts
Source: BMC Microbiol. 2022 Jun 16;22:158. doi: 10.1186/s12866-022-02573-3 (PMC9202165; doi:10.1186/s12866-022-02573-3)
Supplement: Supplementary file 2 — Additional file 2: Table S2. Unique core genes oftwo subspecies of B. catenulatum. [file 12866_2022_2573_MOESM2_ESM.docx]

**Table S2 Unique core genes of two subspecies of *B. catenulatum***

| Unique core genes of *B. catenulatum* subsp. *catenulatum* | Functions | Unique core genes of *B. catenulatum* subsp. *kashiwanohense* | Functions |
| --- | --- | --- | --- |
| group_1001 | Putative bifunctional phosphatase/peptidyl-prolyl cis-trans isomerase | *deg*U | Transcriptional regulatory protein *deg*U |
| *yxe*P | putative hydrolase *yxe*P | *sac*A | Sucrose-6-phosphate hydrolase |
| *fab*G | 3-oxoacyl-[acyl-carrier-protein] reductase *fab*G | *qac*A | Antiseptic resistance protein |
| group_1013 | Acyl-protein synthetase, *lux*E | group_1223 | Putative pyrimidine permease *rut*G |
| *puc*K | Uric acid permease *puc*K | *kst*R2 | HTH-type transcriptional repressor *kst*R2 |
| *ars*C2 | Arsenate-mycothiol transferase *ars*C2 | *cop*R | Transcriptional activator protein *cop*R |
| group_1109 | hypothetical protein | group_1253 | Uracil DNA glycosylase superfamily protein |
| *sil*P | Copper-exporting P-type ATPase A | *yda*F | Putative ribosomal N-acetyltransferase *yda*F |
| group_1169 | SprT-like family protein | *chi*A | putative bifunctional chitinase/lysozyme precursor |
| group_134 | PspC domain protein | group_1286 | hypothetical protein |
| group_1411 | hypothetical protein | group_1287 | *rel*B antitoxin |
| group_1423 | hypothetical protein | group_1295 | hypothetical protein |
| group_1424 | hypothetical protein | group_1298 | Amidohydrolase |
| group_1439 | Transglutaminase-like superfamily protein | group_1304 | Cell division protein *crg*A |
| group_1522 | Sulfatase | *cyt*R | HTH-type transcriptional repressor *cyt*R |
| *mle*N | Malate-2H(+)/Na(+)-lactate antiporter | group_1310 | Acetyltransferase (GNAT) family protein |
| group_1532 | hypothetical protein | group_1312 | Divergent AAA domain protein |
| group_1533 | hypothetical protein | group_1313 | Dihydroorotate dehydrogenase B (NAD(+)), catalytic subunit |
| *gnd*_1 | 6-phosphogluconate dehydrogenase, decarboxylating | group_1314 | hypothetical protein |
| group_1579 | hypothetical protein | *gnd*A | 6-phosphogluconate dehydrogenase, NADP(+)-dependent, decarboxylating |
| group_1595 | TM2 domain protein | group_1319 | Archaeal ATPase |
| *thi*E | Thiamine-phosphate synthase | *lip*2 | Lipase 2 |
| *opp*B | Oligopeptide transport system permease protein *opp*B | group_1323 | hypothetical protein |
| *opp*C | Oligopeptide transport system permease protein *opp*C | group_1324 | hypothetical protein |
| group_1644 | hypothetical protein | group_1325 | hypothetical protein |
| group_1645 | hypothetical protein | group_1326 | Chromosome-partitioning ATPase |
| group_1646 | hypothetical protein | group_1330 | *prg*I family protein |
| group_1647 | Putative peptidoglycan binding domain protein | group_1337 | hypothetical protein |
| group_1649 | hypothetical protein | group_1872 | hypothetical protein |
| *met*I | D-methionine transport system permease protein *met*I | group_1879 | Oligopeptide transport system permease protein *opp*C |
| *met*N2 | Methionine import ATP-binding protein *met*N 2 | group_1880 | Oligopeptide transport system permease protein *opp*B |
| *yvd*D | LOG family protein *yvd*D | group_1881 | Oligopeptide-binding protein *opp*A precursor |
| group_1663 | Acyl-CoA reductase (*lux*C) | *gsi*A | Glutathione import ATP-binding protein *gsi*A |
| group_1664 | putative ABC transporter ATP-binding protein/MT1014 | *gsi*D | Glutathione transport system permease protein *gsi*D |
| *rut*G | Putative pyrimidine permease *rut*G | *dpp*B | Dipeptide transport system permease protein *dpp*B |
| *ydd*G | Aromatic amino acid exporter *ydd*G | *dpp*E | Dipeptide-binding protein *dpp*E precursor |
| *ppx* | Exopolyphosphatase | group_1899 | hypothetical protein |
| *glu*Q | Glutamyl-Q tRNA(Asp) synthetase | group_1900 | L-arabinose transport system permease protein *ara*Q |
| group_1777 | hypothetical protein | *yte*P | putative multiple-sugar transport system permease *yte*P |
| group_1783 | Alpha-galactosidase | *lip*O | Lipoprotein *lip*O precursor |
| group_18 | macrolide transporter ATP-binding /permease protein | *eps*L | putative sugar transferase *eps*L |
| *crg*A | Cell division protein *crg*A | group_1964 | enterobactin exporter *ent*S |
| tpd | 34 kDa membrane antigen precursor | group_1965 | hypothetical protein |
| group_277 | hypothetical protein | group_1974 | hypothetical protein |
| *met*Q | Methionine-binding lipoprotein *met*Q precursor | group_1975 | Lipoprotein-releasing system ATP-binding protein *lol*D |
| group_309 | hypothetical protein | group_1976 | hypothetical protein |
| group_379 | hypothetical protein | group_1977 | hypothetical protein |
| *pyr*DB | Dihydroorotate dehydrogenase B (NAD(+)), catalytic subunit | *cre*C | Sensor protein *cre*C |
| group_420 | hypothetical protein | group_2040 | D-methionine transport system permease protein MetI |
| group_540 | hypothetical protein | *met*N | Methionine import ATP-binding protein *met*N |
| group_550 | major facilitator superfamily transporter | group_2042 | D-methionine-binding lipoprotein *met*Q precursor |
| group_615 | Transposase IS200 like protein | group_2043 | Putative bifunctional phosphatase/peptidyl-prolyl cis-trans isomerase |
| group_619 | hypothetical protein | group_2104 | hypothetical protein |
| group_658 | hypothetical protein | group_2126 | hypothetical protein |
| group_673 | ApbE family protein | *pem*K | mRNA interferase *pem*K |
| group_814 | hypothetical protein | group_2163 | hypothetical protein |
| group_835 | hypothetical protein | group_2164 | hypothetical protein |
| group_895 | Divergent AAA domain protein | group_2168 | L-fuconate dehydratase |
| group_897 | hypothetical protein | group_2169 | 3-alpha-(or 20-beta)-hydroxysteroid dehydrogenase |
| *men*A | 1,4-dihydroxy-2-naphthoate octaprenyltransferase | *yag*E | putative 2-keto-3-deoxy-galactonate aldolase *yag*E |
| group_993 | recombination regulator *rec*X | group_2182 | hypothetical protein |
| group_995 | hypothetical protein | *cyc*A | D-serine/D-alanine/glycine transporter |
| group_996 | hypothetical protein | group_2203 | Aromatic amino acid exporter *ydd*G |
|  |  | group_2219 | 34 kDa membrane antigen precursor |
|  |  | group_2235 | hypothetical protein |
|  |  | group_2236 | Alpha/beta hydrolase family protein |
|  |  | *nai*P | Putative niacin/nicotinamide transporter *nai*P |
|  |  | *rbs*R | Ribose operon repressor |
|  |  | group_2242 | Nucleotidyltransferase domain protein |
|  |  | group_2243 | hypothetical protein |
|  |  | *ser*B | Phosphoserine phosphatase |
|  |  | *ync*A | N-acyltransferase *ync*A |
|  |  | group_2246 | hypothetical protein |
|  |  | group_2256 | TrbL/VirB6 plasmid conjugal transfer protein |
|  |  | group_348 | FtsX-like permease family protein |
|  |  | *pga*C | N-glycosyltransferase |
|  |  | group_370 | hypothetical protein |
|  |  | group_378 | N-acetylmuramoyl-L-alanine amidase domain-containing protein precursor |
|  |  | group_693 | hypothetical protein |
|  |  | group_712 | bifunctional antitoxin/transcriptional repressor *rel*B |
|  |  | group_733 | putative hydrolase *yxe*P |
|  |  | *hdl* IVa | (S)-2-haloacid dehalogenase 4A |
|  |  | *axe*A1 | Acetylxylan esterase precursor |
|  |  | group_760 | hypothetical protein |
|  |  | group_767 | hypothetical protein |
|  |  | group_772 | Putative prophage phiRv2 integrase |
|  |  | group_773 | putative ABC transporter ATP-binding protein *yjj*K |
