# Supplementary material for: Comparative genomic analysis revealed genetic divergence between Bifidobacterium catenulatum subspecies present in infant versus adult guts
Source: BMC Microbiol. 2022 Jun 16;22:158. doi: 10.1186/s12866-022-02573-3 (PMC9202165; doi:10.1186/s12866-022-02573-3)
Supplement: Supplementary file 3 — Additional file 3: Table S3. Information on HMOgene clusters of B. catenulatum subsp. kashiwanohenseand its references. [file 12866_2022_2573_MOESM3_ESM.docx]

**Table S3** Information on HMO gene clusters of *B. catenulatum* subsp. *kashiwanohense* and its references.

| Strains | Range | Size (bp) | GC content (%) |
| --- | --- | --- | --- |
| *B. longum* subsp. *longum* SC596 | BLNG_01264-01254 | 12,985 | 55.06 |
| *B. pseudocatenulatum* JCM1200^T^ | BBPC_1770-1778 | 11,065 | 55.16 |
| *B. catenulatum* subsp. *kashiwanohense* JCM15439^T^ | BBKW_1831-1841 | 12,998 | 53.84 |
| *B. catenulatum* subsp. *kashiwanohense* APCKJ1 | BKKJ1_2069-2079 | 12,998 | 53.88 |
| *B. catenulatum* subsp. *kashiwanohense* DSM21854^T^ | BKAS_1814-1804 | 12,998 | 53.84 |
| *B. catenulatum* subsp. *kashiwanohense* DSM21854(2)^T^ | OU18_RS08880-08930 | 12,998 | 53.84 |
| *B. catenulatum* subsp. *kashiwanohense* PV20-2 | AH68_10220-10260 | 11,065 | 54.98 |
