# Supplementary figures and images for: Comparative genomic analysis revealed genetic divergence between Bifidobacterium catenulatum subspecies present in infant versus adult guts
Source: BMC Microbiol. 2022 Jun 16;22:158. doi: 10.1186/s12866-022-02573-3 (PMC9202165; doi:10.1186/s12866-022-02573-3)

A

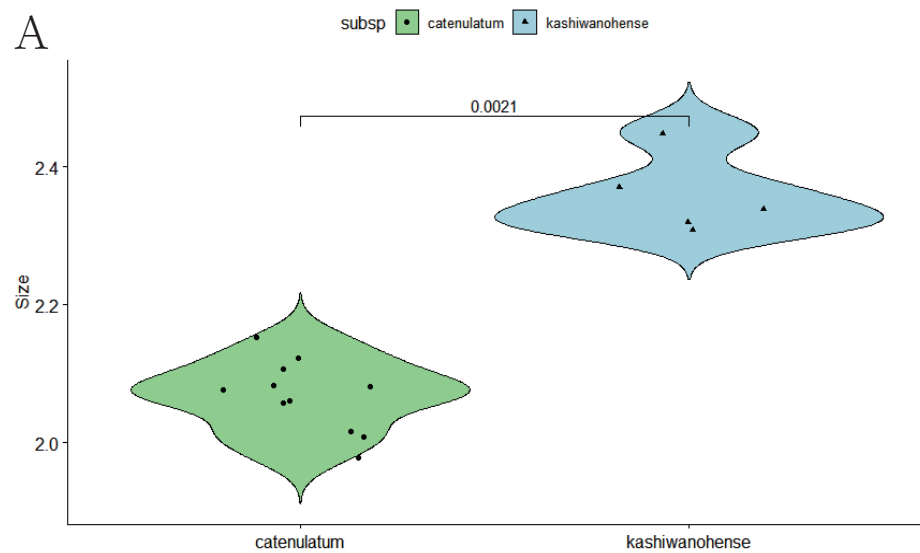

Supplement: Supplementary file 4 — Additional file 4: Fig. S1. Comparison ofgenomic features between two subspecies of B. catenulatum, includinggenome size (A), GC Content (B), CDSs (C) and tRNA (D). [file 12866_2022_2573_MOESM4_ESM.pdf]

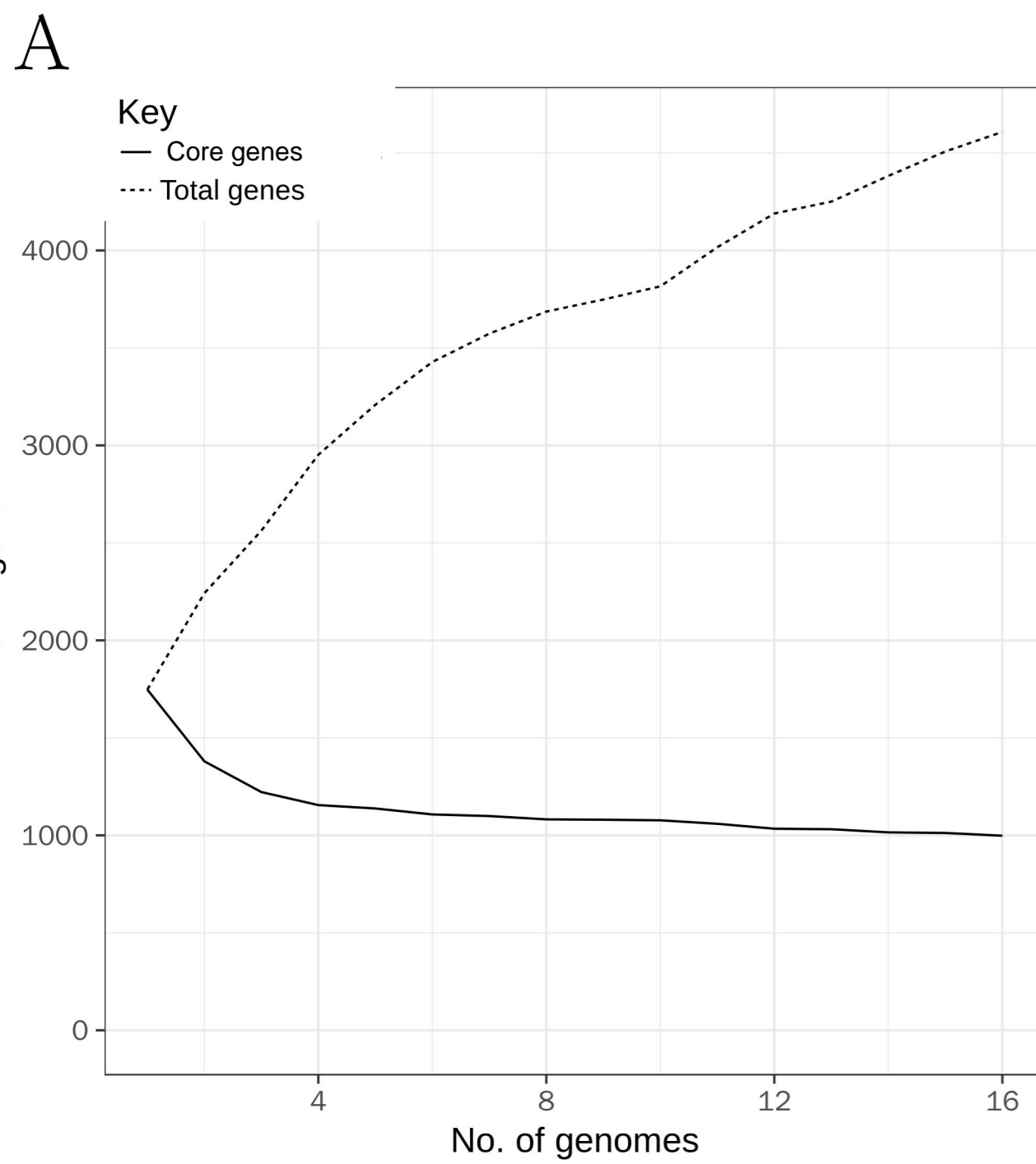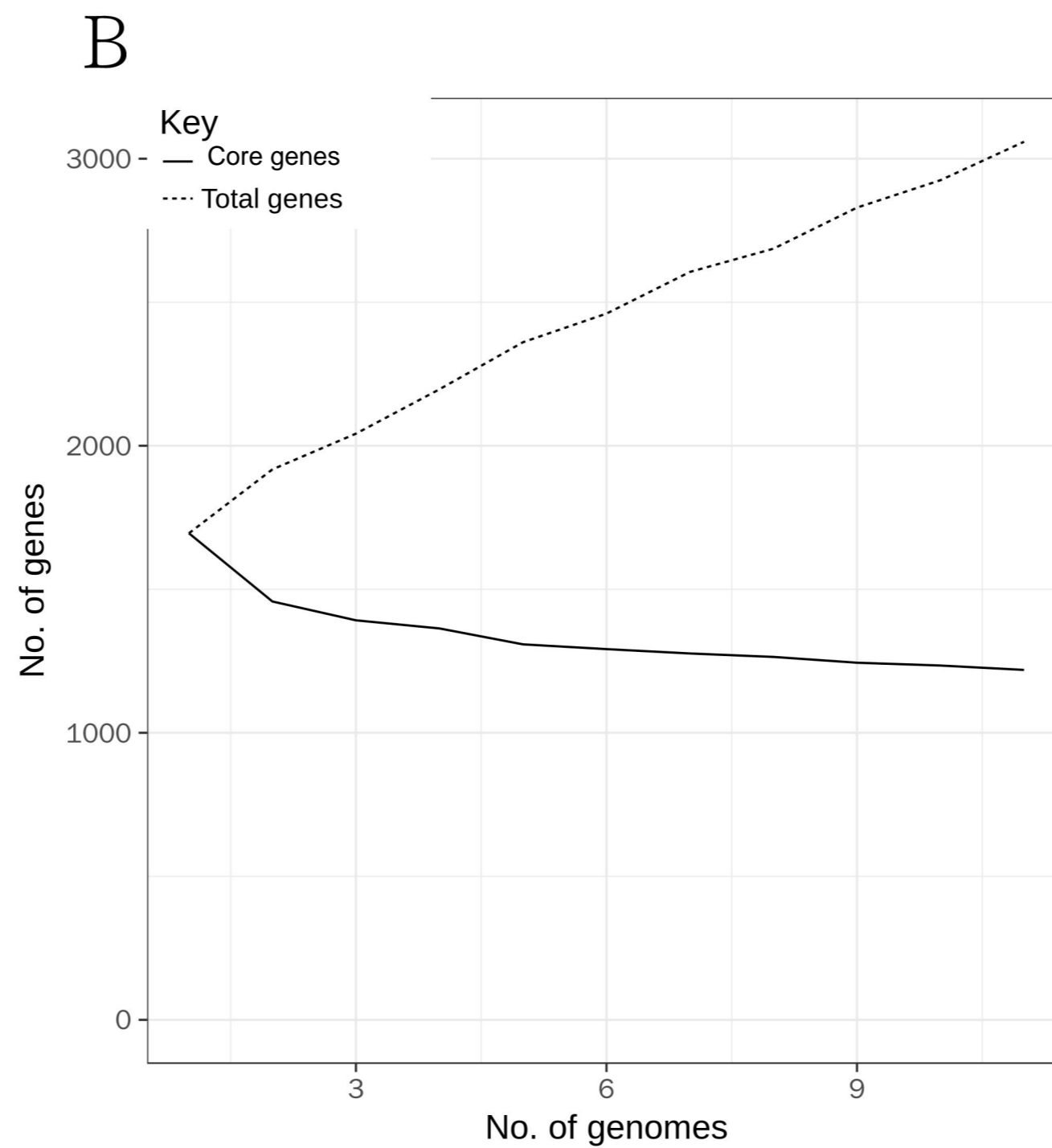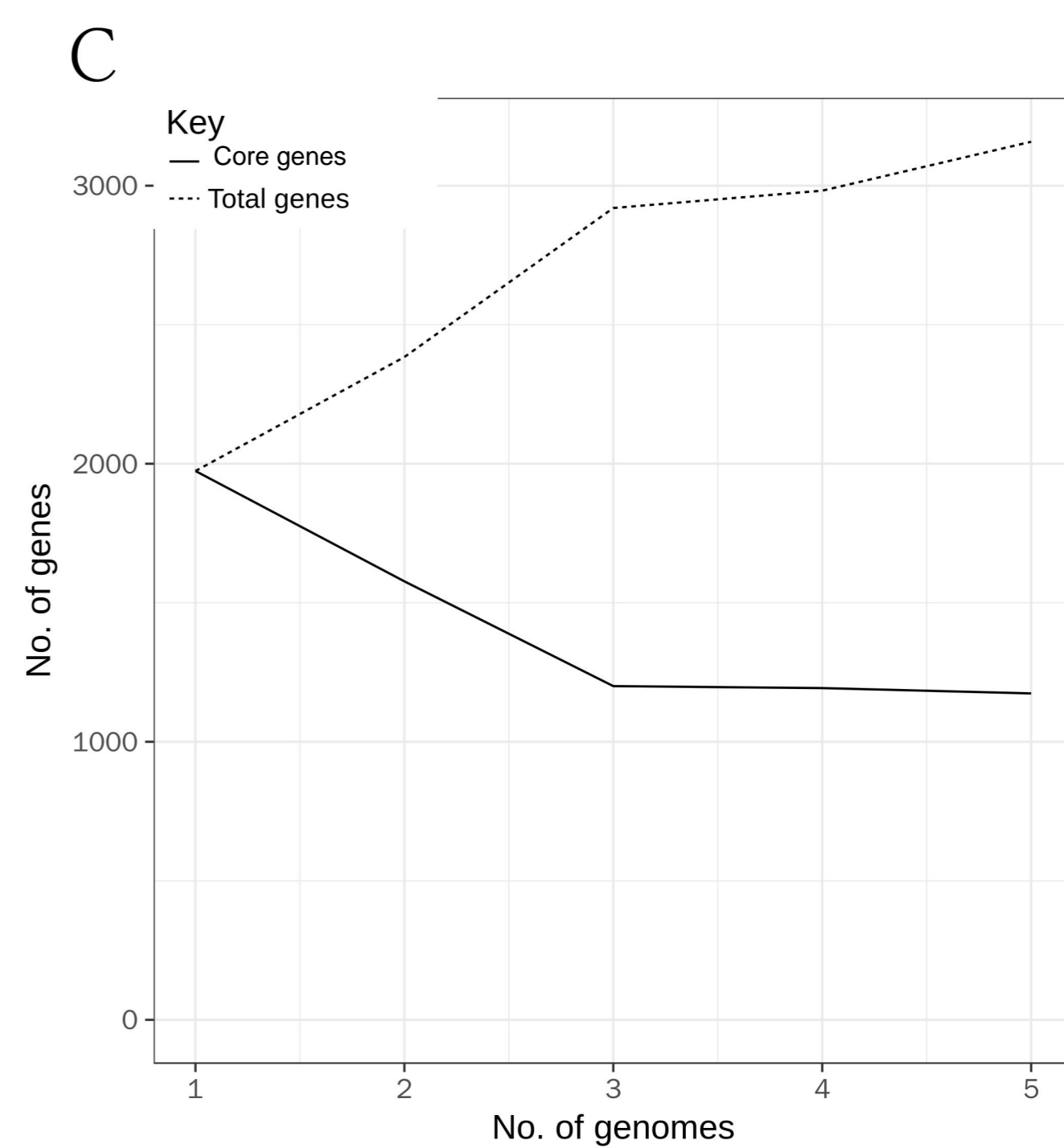

Supplement: Supplementary file 6 — Additional file 6: Fig. S3. Tendency curves forpan-core genomes of B. catenulatum. B. catenulatum (A), B.catenulatum subsp. catenulatum (B), B. catenulatumsubsp. kashiwanohense (C). [file 12866_2022_2573_MOESM6_ESM.pdf]

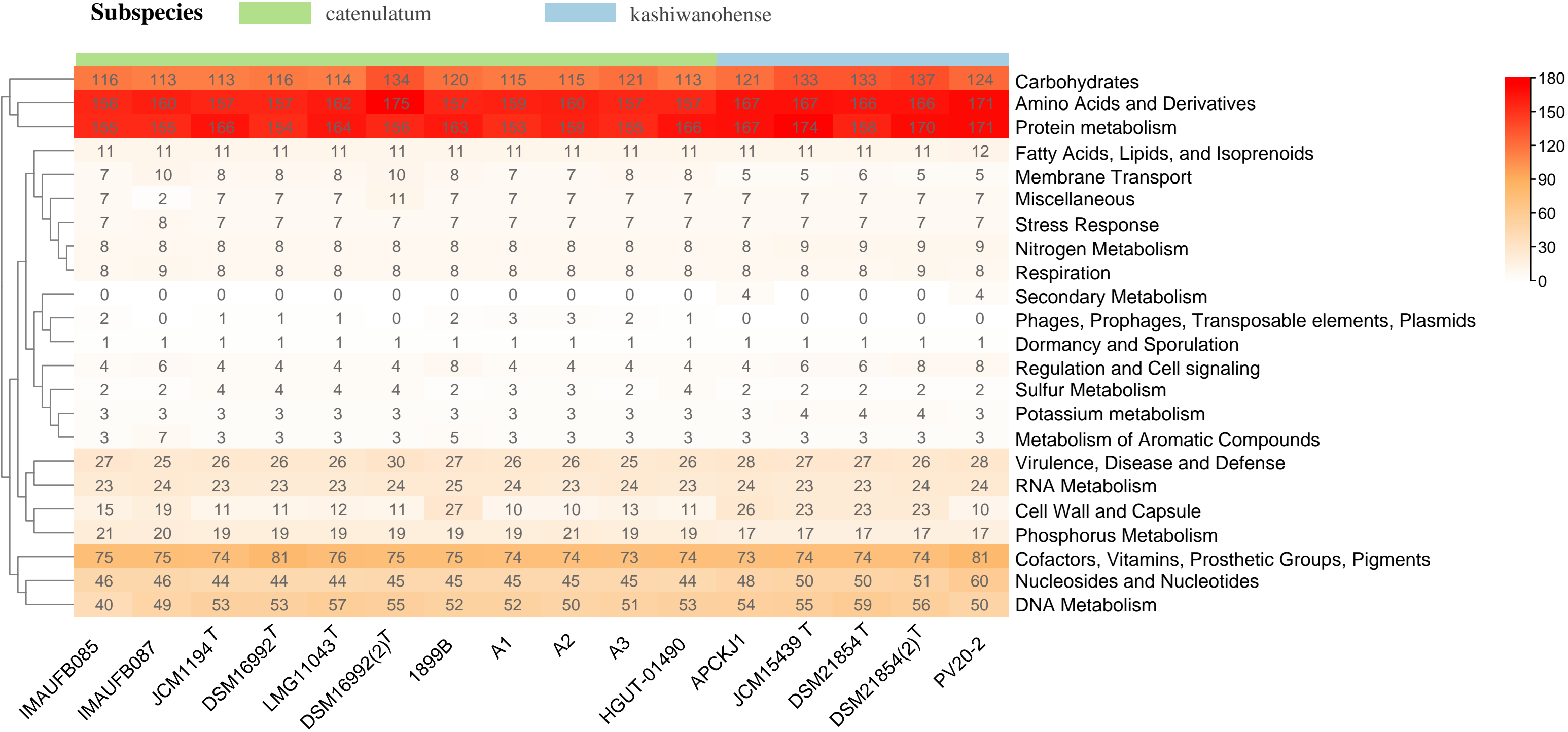

Supplement: Supplementary file 7 — Additional file 7: Fig. S4. Comparison of 23functional categories between B. catenulatum genomes. The numbers in theheat map indicate the number of copies of functional genes. [file 12866_2022_2573_MOESM7_ESM.pdf]

group ■ full\_length ▲ HMO\_cluster

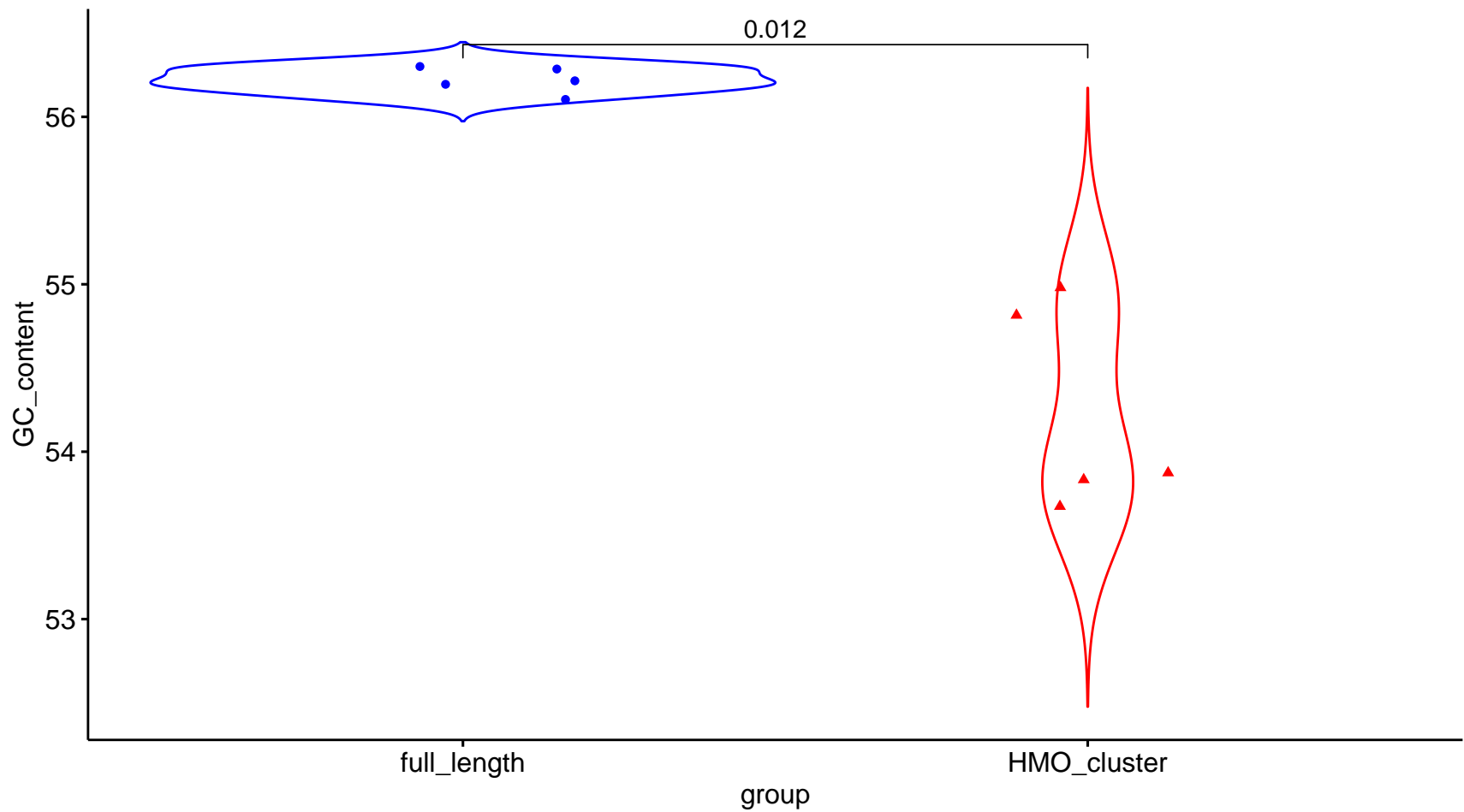

Supplement: Supplementary file 8 — Additional file 8: Fig. S5. Comparisonof GC content between full-length genomes and FHMO clusters in B. catenulatumsubsp. kashiwanohense. [file 12866_2022_2573_MOESM8_ESM.pdf]
