# Supplementary material for: Comparative genomic analysis revealed genetic divergence between Bifidobacterium catenulatum subspecies present in infant versus adult guts
Source: BMC Microbiol. 2022 Jun 16;22:158. doi: 10.1186/s12866-022-02573-3 (PMC9202165; doi:10.1186/s12866-022-02573-3)
Supplement: Supplementary file 5 — Additional file 5: Fig. S2. A display of thegenome circle map of 16 B. catenulatum genomes. The figure was generatedbased on comparison of 16 B. catenulatum genomes according to percentageidentity (100%, 90%, or 70%). The numbers on the rings from inside to outside,1: JCM15439T, 2: DSM21854(2)T, 3: DSM21854T,4: APCKJ1, 5: PV20-2, 6: HGUT-01490, 7: LMG11043T, 8: A1, 9: A3, 10:A2, 11: 1899B, 12: DSM16992T, 13: DSM16992(2)T, 14:JCM1164T, 15: IMAUFB085, 16: IMAUFB087. [file 12866_2022_2573_MOESM5_ESM.pdf]

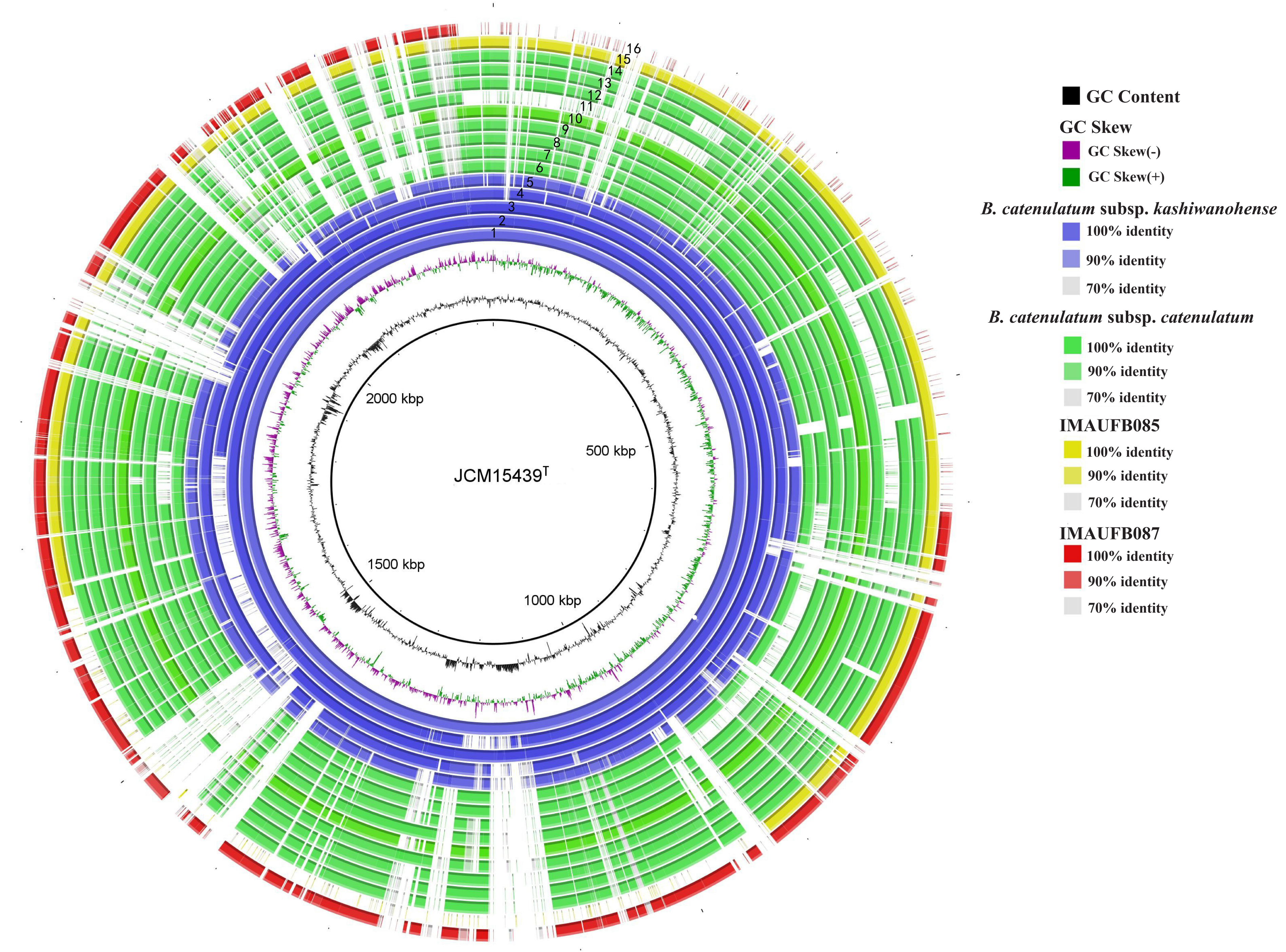

JCM15439T
500 kbp
1000 kbp
1500 kbp
2000 kbp
1
2
3
4
5
6
7
8
9
10
11
12
13
14
15
16
GC Content
GC Skew
GC Skew(-)
GC Skew(+)
B. catenulatum subsp. kashiwanohense
100% identity
90% identity
70% identity
B. catenulatum subsp. catenulatum
100% identity
90% identity
70% identity
IMAUFB085
100% identity
90% identity
70% identity
IMAUFB087
100% identity
90% identity
70% identity

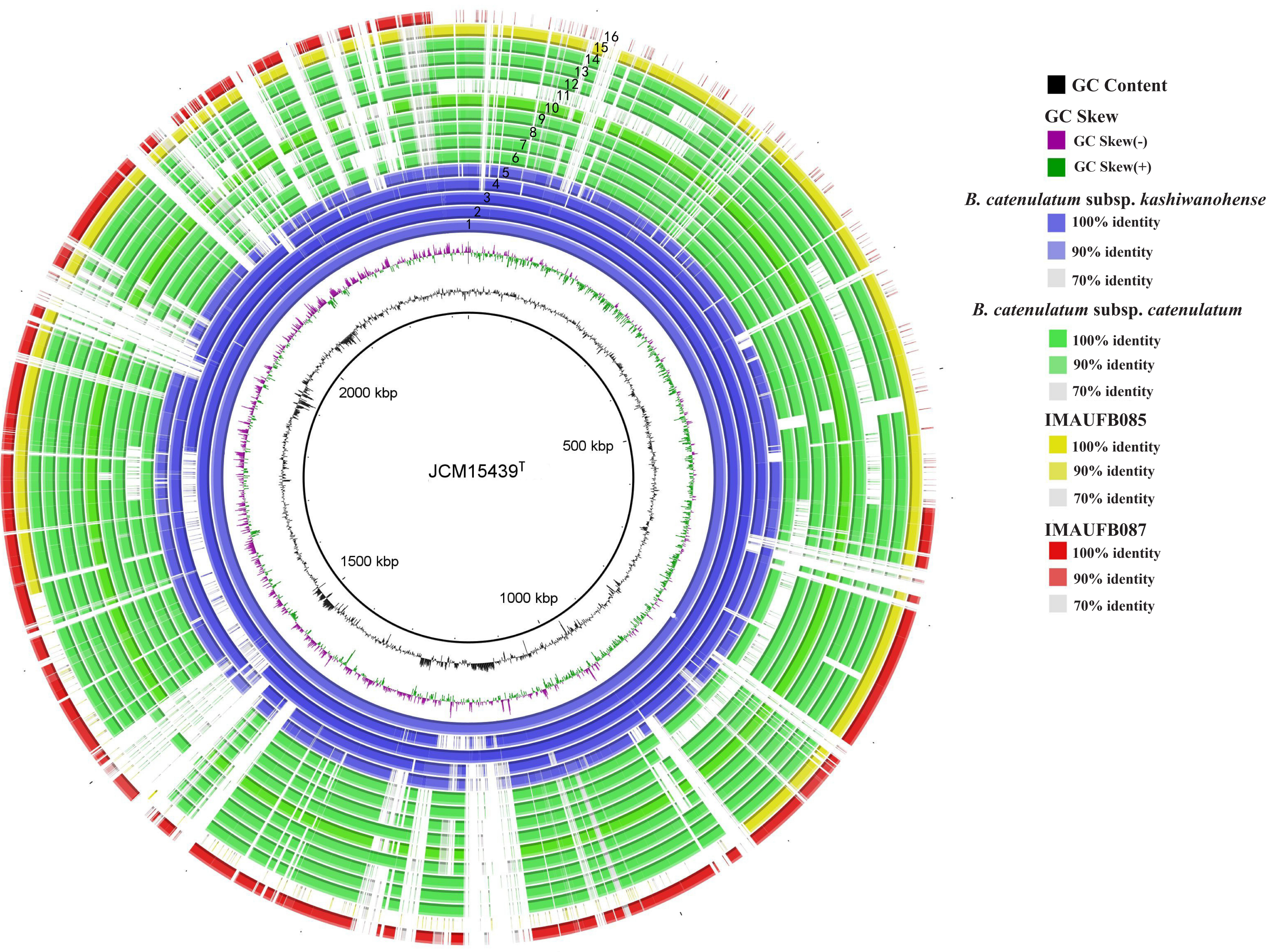

JCM15439T
500 kbp
1000 kbp
1500 kbp
2000 kbp
1
2
3
4
5
6
7
8
9
10
11
12
13
14
15
16
GC Content
GC Skew
GC Skew(-)
GC Skew(+)
B. catenulatum subsp. kashiwanohense
100% identity
90% identity
70% identity
B. catenulatum subsp. catenulatum
100% identity
90% identity
70% identity
IMAUFB085
100% identity
90% identity
70% identity
IMAUFB087
100% identity
90% identity
70% identity
